# Increasing Diversity in Cardiology: A Fellowship Director’s Perspective

**DOI:** 10.7759/cureus.16344

**Published:** 2021-07-12

**Authors:** Amman Bhasin, Arif Musa, Louis Massoud, Azar Razikeen, Arshia Noori, Ali Ghandour, David Gelovani, Luis C Afonso, Randy Lieberman, Ajay Vaidya

**Affiliations:** 1 Internal Medicine, Wayne State University School of Medicine, Detroit, USA; 2 Radiology, Wayne State University School of Medicine, Detroit, USA; 3 Family Medicine, Wayne State University School of Medicine, Detroit, USA; 4 Cardiology, Cedars-Sinai Smidt Heart Institute, Los Angeles, USA; 5 Internal Medicine, Henry Ford Health System, Detroit, USA; 6 Cardiology, Wayne State University, Detroit, USA; 7 Cardiology, Keck School of Medicine, Los Angeles, USA

**Keywords:** diversity, under-represented minority, graduate medical education, cardiology, fellowship

## Abstract

Background

Underrepresented-minorities (URM) remain few in number amongst practicing cardiologists and across cardiology fellowship training programs in the U.S. Increased diversity is needed across the entire field and is particularly necessary within graduate medical education cardiology fellowship training programs.

Objectives

This cross-sectional study was performed to identify which strategies were supported and implemented by cardiology fellowship program directors (PDs) to increase URM representation, to determine which entities hold the responsibility to increase diversity according to program directors, and to quantify URM representation in cardiology fellowship programs.

Methods

A 15-item survey was submitted to all American College of Graduate Medical Education (ACGME) accredited cardiology fellowship programs via electronic mail.

Results

Of 250 cardiology fellowship programs, 71 responses were received (28.4%). The number of URM faculty varied from 0-1 to more than six, and URM faculty held leadership roles in most programs (62.0%). A total of 16 respondents (22.5%) were URM program directors. Most respondents agreed that diversity was important to their training program (85.9%). The majority endorsed direct recruitment of URM applicants (60.6%), opportunities for applicants to connect with (54.9%) or be recruited by URM fellows (54.9%), holistic application review (67.6%), promoting mentorship by URM faculty (69.0%), URM faculty involvement in applicant interviewing (54.9%), and increased recruitment of URM faculty members (73.2%). Program directors allocated major responsibility to increase diversity to fellowship programs (71.8%), residency programs (63.3%), and medical schools (53.5%).

Conclusions

This study found that most cardiology programs have URM faculty in leadership roles, and nearly a quarter of the surveyed program directors were URMs. Cardiology program directors endorsed and employed numerous strategies to increase diversity and URM representation in fellowship programs. Additionally, program directors held fellowship training programs most responsible for increasing URM representation in the field of cardiology.

## Introduction

Underrepresented-minority (URM) physicians, specifically African American, Native American, Hispanic, and/or Pacific Islander, constitute only about 10% of practicing cardiologists [1-3]. Diversity and inclusion in the workplace have been widely accepted as important factors that optimize organizational outcomes as they have been shown to improve innovation, increase financial performance, and maximize productivity [4-5]. Increased diversity in medical fields has been shown to contribute to improved overall patient experience, clinical care decisions, and quality of patient care [4-5]. Despite the advantages of having a diverse physician workforce, the field of cardiology has failed to keep pace with the increasingly diverse patient population of the United States [6]. In fact, 30% of cardiovascular training programs accredited by the American College of Graduate Medical Education (ACGME) had no URMs, and over half had only between 1% and 25% of fellows that comprised that category [7]. Underrepresentation has not gone unnoticed by the broader physician community, as evidenced by the 2019 establishment of workforce diversity guidelines by the ACGME, which have been described as a “wake-up call” to cardiology [6]. Moreover, the 2020 retraction of an article published by a cardiology fellowship program director due to numerous misconceptions, inaccuracies, and other invalidities regarding diversity underscores the necessity of continued research in this subject [8].

A review of data from the past decade has shown progress in increasing the number of cardiology trainees who would be considered URMs. However, URM cardiologists remain a considerable minority in the cardiologist workforce nationally. In fact, the percentage of URM cardiologists increased only modestly from 11.1% to 12.4% from 2006 to 2016, respectively [9]. In addition, there are cardiology fellowship programs with decades of existence that have never trained a URM fellow until recently [10].

Given that fellowships constitute the entryway into the field of cardiology, the role of the fellowship program director (PD) in increasing diversity in the cardiologist workforce cannot be overstated. A 2016 survey by Crowley et al. reported that 63% of fellowship program directors believed that diversity in their program was adequate and did not require further intervention; this study also reported that 69% of the same respondents agreed with the statement, “diversity is a driver of excellence in healthcare delivery” [11]. Another survey of program directors by Damp et al. described cardiology program culture, lack of support for URM trainees, and lack of diversity among current fellows as major barriers to increasing diversity [12].

This survey of cardiology fellowship program directors builds upon previous research in several unique ways. This study was structured to quantify URM applicants, fellows, and faculty members in ACGME-accredited cardiology fellowship programs and identify which strategies are supported and currently implemented by program directors. Moreover, this study seeks to determine which entities are viewed by fellowship directors to have a role in increasing diversity in cardiology training programs.

## Materials and methods

Fellowship program database construction

The University of Southern California Biomedical Institutional Review Board (BioIRB) determined this study to be exempt (#HS-20-00319). After receiving this IRB approval, we conducted an anonymous cross-sectional study of 250 ACGME-accredited cardiology fellowship program directors in the United States. The following data were collected: program name and location, director full name, program director email address, and program coordinator email address. Data were abstracted from the Fellowship & Residency Electronic Interactive Database (FREIDA) and cross-referenced with the ACGME website. If the program director's contact information was unavailable, we attempted to obtain this information through internet searches of faculty directories, corresponding author contact information (e.g., original research articles), and scientific databases (e.g., PubMed).

Survey development and dissemination

Survey questions were created through an iterative process by members of the research team & diversity content experts and by referencing previous surveys created to assess diversity in medical fellowship programs [13]. The survey was presented at institutional and local conferences to obtain feedback from research faculty and diversity content experts about content validity. The final questionnaire consisted of 15 items. Within the questionnaire, an underrepresented minority (URM) was defined as any individual whose racial or ethnic identity is: Hispanic, Black, American Indian, Native Alaskan, Native Hawaiian, or Native Pacific Islander [11]. Items one through five and item nine assessed fellowship program demographic data (region, location, length of program, number of fellowship positions, and number of applicants interviewed). Items six through eight and 10-11 assessed URM demographic information for each program (number of URM fellows, matriculants, interviews, faculty, leaders, and program directors). Items 12-15 assessed respondent views regarding the importance of diversity, strategies to increase diversity, and responsibility to increase diversity. The full questionnaire is provided in the Appendices. Google Forms (Google Inc., Mountain View, CA) was used to obtain responses from study participants. Data were collected from September to December 2020. A total of three reminder emails were submitted to cardiology fellowship directors. A lottery incentive was used to increase the response rate.

Statistical analysis

Descriptive statistics were applied to the dataset. Fisher’s exact tests were used to compare the number of respondents that supported each strategy to increase diversity to respondents that actively implemented those measures. Predictors of strategies implemented by PDs to increase diversity were identified a priori: URM status and agreement with the statement “diversity is important to my residency program.” We then dichotomized agreement regarding the importance of diversity into two variables prior to comparison: PDs that “strongly agreed” versus PDs that “somewhat agreed,” “neither agree nor disagreed,” “somewhat disagreed,” and “strongly disagreed” with the above statement. The latter group was hereby referred to as “not strongly agreed” in ensuing data comparisons. Fisher’s exact tests were used to (1) compare strategies that were implemented by respondents who identified themselves as a URM to respondents who identified themselves as non-URM members, and (2) compare strategies that were implemented by PDs that “strongly agreed” versus those who did “not strongly agree.” P-values less than 0.05 were considered statistically significant.

## Results

A total of 71 responses were obtained from 250 ACGME-accredited cardiology fellowship training programs, constituting a 28.4% response rate. The total number of fellowship training positions available ranged from 3 to 35, with an average of 15.3 positions per program (standard deviation (SD) = 6.8). Cardiology fellowship training program characteristics are described in Table 1.

**Table 1:** Cardiology Fellowship Training Program Characteristics and URM Representation URM: underrepresented minority - defined as any individual whose racial or ethnic identity is: Hispanic, Black, American Indian, Native Alaskan, Native Hawaiian, or Native Pacific Islander. Non-URM: an individual who is not defined as an underrepresented minority member. *Hybrid = Community-Based/University Affiliated program as per the FREIDA program type categorization.

Category		n	%
Country Region	West	6	8.9
	Southwest	7	9.9
	Southeast	10	14.11
	Midwest	17	23.9
	Northeast	31	43.7
Program Type	University	53	74.0
	Community	6	8.5
	Hybrid*	12	16.9
Program Location	Urban	59	70.4
	Suburban	19	26.8
	Rural	2	2.8
Program Length	3 years	68	95.8
	4 years	3	4.2
Applicants Reviewed	< 10	0	0.0
	10-20	5	7.2
	20-30	4	5.6
	30-40	6	8.5
	40-50	12	16.9
	> 50	55	77.5
Program Director Demographic	URM	16	22.5
	Non-URM	55	77.5
URM Fellows in Entire Program	0	8	11.3
	1-3	24	33.8
	4-6	27	38.0
	>6	12	16.9
URM Fellows Matriculated this Year	0	20	28.2
	1	25	35.2
	2	16	22.5
	>3	10	14.1
URM Faculty Members in Program	0-1	20	20.8
	2-3	18	25.4
	4-5	19	26.8
	>6	14	19.7
Characteristics of Faculty Leadership	Programs with URM Faculty in Leadership Roles	44	62.0
	Programs with No URM Faculty in Leadership Roles	25	35.2

URM representation in cardiology fellowship programs

The number of URM fellows ranged from 0 to 20, with an average of 4.5 URM fellows per program (SD = 3.8). Of interviewed applicants, the number of URM matriculants ranged from 0 to 4, with an average of 1.3 (SD = 1.1). The reported number of URM faculty per program included 0-1 (n = 20, 28.2%), 2-3 (n = 18, 25.3%), 4-5 (n = 19, 26.8%), and 6 or more (n = 14, 18.7%). URM faculty members reportedly held leadership roles in the majority of fellowship programs (n = 44, 62.0%), although respondents from two programs were unsure if URM faculty held leadership roles (2.8%). Of 71 program directors, 22.5% (n = 16) considered themselves URM. A detailed analysis of URM representation in cardiology fellowship programs is described in Table 1. The majority of respondents strongly agreed (n = 49, 69.0%) or somewhat agreed (n = 12, 16.9%) that diversity was important to their fellowship training program. Of the remaining respondents, 4.2% neither agreed nor disagreed (n = 3), and 8.5% somewhat or strongly disagreed (n = 6) that diversity was of importance.

Outreach strategies to URM residents

Respondents’ support of strategies to increase fellowship diversity and current, active implementation of outreach to URM residents is described in Table 2. Approximately half of program directors endorsed (n = 43, 60.6%) and implemented (n = 35, 49.3%) direct recruitment of URM applicants. Allowing opportunities for URM resident applicants to connect with URM cardiology fellows was supported (n = 42, 59.2%) and actively encouraged (n = 39, 54.9%) by most fellowship directors. Similarly, most program directors supported the involvement of URM fellows in the recruitment of URM resident applicants (n = 39, 54.9%) and implemented such initiatives (n = 44, 62.0%). Fewer respondents endorsed recruiting URM medical students (n = 11, 15.5%) and even fewer were involved in actively recruiting URM medical students to their fellowship programs (n = 2, 2.8%). Also, few respondents supported using stipend or travel funds for outreach to URM applicants (n = 8, 11.3%) and none of the fellowship program directors surveyed recruited applicants by offering funding for travel expenses. In most cases, program directors supported strategies that were actively being implemented in their fellowship programs; however, in two instances, there was a statistically significant difference between the number of program directors who supported versus implemented the following outreach measures: direct recruitment of URM medical students (p = 0.017) and allocating travel funds for URM applicants (n = 0.006). In this instance, program directors supported these strategies without actively pursuing these strategies for implementation in their fellowship program.

**Table 2:** Strategies Involving Outreach to URM Residents Supported and Implemented by Cardiology Fellowship Directors URM: underrepresented minority - defined as any individual whose racial or ethnic identity is: Hispanic, Black, American Indian, Native Alaskan, Native Hawaiian, or Native Pacific Islander. *: p-value < 0.05, indicating statistical significance, p-values obtained via Fisher’s exact test

Strategy	Supported		Implemented		p-value
	n	%	n	%	
Directly recruit URM residents	43	60.6	35	49.3	0.238
Opportunities to connect with URM fellows	42	59.2	39	54.9	0.735
Directly recruit URM medical students	11	15.5	2	2.8	0.017*
Involve fellows to recruit URM residents	39	54.9	44	62.0	0.496
Stipend or travel awards for URM residents	8	11.3	0	0.0	0.006*

Modifications of URM application review

Strategies to address diversity through changes to the application process that are supported by and implemented by cardiology fellowship directors are indicated in Table 3. Most respondents endorsed (n = 48, 67.6%) and implemented (n = 49, 69.0%) conducting a holistic review of cardiology fellowship applicants. Fewer program directors supported URM representation in fellowship application reviewer committees (n = 29, 40.8%) and required structuring review committees in this way (n = 19, 26.8%). Approximately one-third of program directors agreed with deemphasizing United States Medical Licensing Examination (USMLE) scores (n = 24, 33.8%), and a similar number deemphasized scores when reviewing applications (n = 21, 29.6%). Removing photos and ethnicity data from fellowship applications was supported (n = 16, 22.5%) and implemented (n = 11, 15.5%) by a minority of respondents. Few fellowship directors endorsed (n = 16, 22.5%) and reviewed (n = 9, 12.7%) more fellowship applications from international medical graduates to increase URM representation. Most program directors did not support (n = 7, 9.9%) or establish (n = 5, 7.0%) a minimum number of ranked URM applicants in the creation of their match list.

**Table 3:** Strategies Involving Altering Application Review Supported and Implemented by Cardiology Fellowship Directors URM: underrepresented minority - defined as any individual whose racial or ethnic identity is: Hispanic, Black, American Indian, Native Alaskan, Native Hawaiian, or Native Pacific Islander. *: p-value < 0.05, indicating statistical significance, p-values obtained via Fisher’s exact test

Strategy	Supported		Implemented		p-value
	n	%	n	%	
Deemphasize USMLE scores	24	33.8	21	29.6	0.719
Conduct holistic application review	48	67.6	49	69.0	1.000
Remove photos and ethnicity labels	16	22.5	11	15.5	0.393
Require a number of URM acceptances	7	9.9	5	7.0	0.764
Require URMs in applicant review committee	29	40.8	19	26.8	0.110
Review more international medical graduates	16	22.5	9	12.7	0.186

The use of diversity-related programming

The numbers and percentages of fellowship directors that support and implement various diversity-related programming are described in Table 4. Most program directors supported (n = 49, 69.0%) and implemented (n = 43, 60.6%) initiatives to promote support and mentoring of URM applicants by URM faculty. Most fellowship program directors also supported increasing URM faculty involved in interviewing applicants (n = 39, 54.9%) and a large proportion included URM interviewers during applicant interviews (n = 33, 46.5%). Fewer respondents supported the recruitment of URM applicants after the interview (n = 30, 42.3%) or directly recruiting URM applicants following the interview (n = 27, 38.0%).

**Table 4:** Strategies Involving Diversity Related Programming Supported and Implemented by Cardiology Fellowship Directors URM: underrepresented minority - defined as any individual whose racial or ethnic identity is: Hispanic, Black, American Indian, Native Alaskan, Native Hawaiian, or Native Pacific Islander. *: p-value < 0.05, indicating statistical significance, p-values obtained via Fisher’s exact test

Strategy	Supported		Implemented		p-value
	n	%	n	%	
Promote URM faculty mentoring and support	49	69.0	43	60.6	0.380
Increase URM faculty conducting interviews	39	54.9	33	46.5	0.401
Directly recruit URM applicants after interview	30	42.3	27	38.0	0.732

Support systems for URM fellows & diversity initiatives

Strategies of active promotion of diversity within cardiology fellowship training programs according to program directors are described in Table 5. Most respondents supported the increased recruitment of URM faculty members (n = 52, 73.2%) and most actively recruited URM faculty for their training programs (n = 45, 63.4%). Fewer program directors endorsed allocating additional funds for increasing diversity (n = 31, 43.7%) and about 30% reported increasing funding in their programs (n = 21, 29.6%). A minority of respondents reported supporting formally addressing abuse of URM fellows (n = 31, 43.7%) or implementing such formalized programs (n = 25, 21.1%). Establishing URM small groups for the purposes of discussing workplace discrimination was not supported (n = 19, 26.8%) or implemented (n = 14, 19.7%) by most fellowship directors.

**Table 5:** Strategies Involving Actively Promoting Diversity Supported and Implemented by Cardiology Fellowship Directors URM: underrepresented minority - defined as any individual whose racial or ethnic identity is: Hispanic, Black, American Indian, Native Alaskan, Native Hawaiian, or Native Pacific Islander. *: p-value < 0.05, indicating statistical significance, p-values obtained via Fisher’s exact test

Strategy	Supported		Implemented		p-value
	n	%	n	%	
Increase funding for diversity initiatives	31	43.7	21	29.6	0.117
Increase URM faculty members	52	73.2	45	63.4	1.000
Establish URM groups for workplace issues	19	26.8	14	19.7	0.427
Formalize reporting/addressing URM abuse	31	43.7	25	21.1	0.391

Comparing responses of URM program directors vs. non-URM program directors

Of 71 program director respondents, 22.5% (n = 16) identified themselves as URM members, while 77.5% (n=55) identified themselves as non-URM members. A statistically significant difference was found regarding the involvement of current program fellows in informal recruitment of URM applicants (p =0.0007) as 70.9% (n=39) of non-URM program directors implemented this strategy compared to only 31.3% (n=5) of URM program directors. No other statistically significant differences were found in the implementation of any other strategies between URM PDs compared to non-URM PDs (p > 0.05).

Implementation strategies of program directors that “strongly agree” vs. “not strongly agree” that diversity is important to their fellowship program

Comparative responses of diversity strategies that are currently being implemented by respondents who responded as “strongly agreed” (n=49) to the statement, “diversity is important to my residency program,” were contrasted against program directors who responded with any other response (n=22) (“not strongly agree”). Despite similarities between the groups, a statistically significant difference was found between the groups when implementing a holistic review of URM fellowship applicants (p = 0.028) as program directors that “strongly agree” with the importance of diversity implemented this strategy at a rate of 77.6% (n=38), while only 50.0% (n=11) of program directors that placed lesser importance on diversity implemented this strategy. No other statistically significant differences were found in the implementation of any other strategies between “strongly agree” PDs compared to “not strongly agree” PDs (p > 0.05).

Whose responsibility is it to increase diversity in cardiology fellowships?

**Figure 1:**
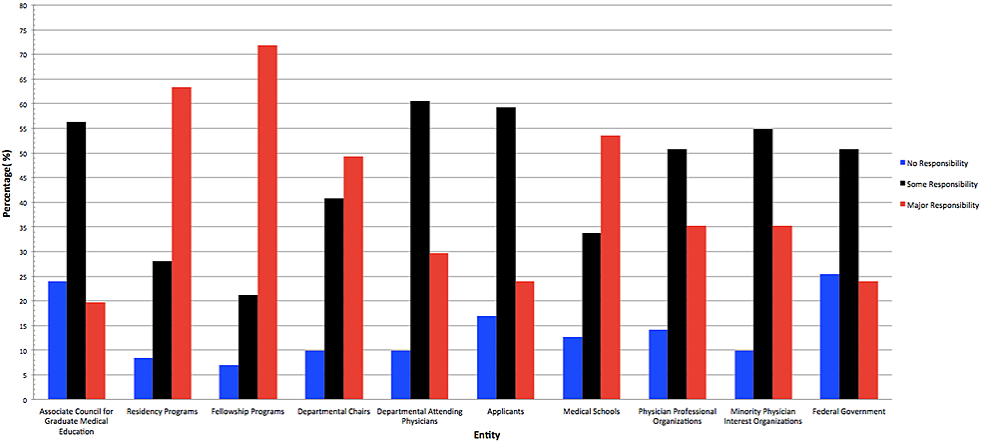
Entities with Responsibility to Increase Diversity in Cardiology Programs According to Cardiology Program Directors

Responsibility for increasing diversity according to fellowship program directors is described in Figure 1. Most fellowship directors allocated major responsibility to themselves (71.8%), residency programs (63.3%), and medical schools (53.5%). Nearly half of survey respondents allocated major responsibility to departmental chairs (49.3%). Most respondents allocated some responsibility to the departmental faculty (60.6%), applicants (59.2%), ACGME (56.3%), minority physician interest organizations (e.g., National Hispanic Medical Association) (54.9%), the federal government (50.7%), or physician professional organizations (e.g., American College of Cardiology) (50.7%).

## Discussion

This cross-sectional study was performed to assess the strategies endorsed and implemented by cardiology fellowship training program directors and the representation of URM applicants and faculty in ACGME-accredited programs. This study found that the vast majority of PDs expressed agreement with the view that diversity is important in fellowship training programs (85.9%). However, PDs remain unsure how to implement the desire for diversity in the process of selecting candidates and creating a rank list [12]. In fact, a previous survey of fellowship directors showed that only 6% of respondents considered diversity to be a top-three priority when forming the rank list [11].

Furthermore, this study showed that PDs tended to support several different methods to increase URM representation and implemented these strategies to increase diversity in their program. These findings suggest that program directors believe that they have the means to employ methods that meaningfully increase diversity within their programs. As in previous studies, most fellowship directors claimed to support diversity, although a previous study found that less than half of directors reported having a formalized plan to achieve this goal [11-12]. Achieving meaningful increases in diversity in the field of cardiology may require fellowship program directors to identify and implement such strategies.

In order to achieve a higher degree of diversity and inclusion, cardiology fellowship directors largely expressed support for increasing the number of URM faculty members (73.2%). It has previously been shown that the presence of URM faculty, mentorship of URM faculty with URM fellows, and communication between URM faculty and fellows with URM applicants on interview day is critical to recruiting competitive, diverse cardiology fellows [10]. In our survey, most program directors reported involving current fellows in the recruitment process and creating opportunities for URM applicants to connect with active fellows. The majority of program directors also supported holistic application review, increasing the number of URM faculty conducting interviews, and promoting peer support from URM faculty.

Implementation of diversity strategies by URM program directors was similar compared to that of non-URM directors, with the exception of involving current program fellows in the informal recruitment of URMs. Non-URM members overwhelmingly implemented this strategy at a rate of 70.9% (n=39) compared to only 31.3% (n=5) URM program directors. As previously described by Auseon et al., having URM leadership in cardiology fellowship programs is often a “rate-limiting step” to attracting URM fellows to a program [10]. As such, cardiology fellowship programs that have a URM program director may be perceived differently by minority applicants. As a result, non-URM program directors may consider encouraging current fellows to provide personal accounts of program diversity and culture in order to attract URM applicants that prioritize diversity in their fellowship selection.

Program directors that “strongly agreed” that diversity is important to their program implemented diversity strategies at similar rates compared to program directors who did “not strongly agree” with the statement. One notable exception was noted with the implementation of conducting a holistic review of all applicants. More than three-fourths of “strongly agree” respondent program directors implemented this method compared to only half of “not strongly agree” respondents. A previous study conducted within an internal medicine residency program found that a holistic review of applicants increased the percentage of URM applications reviewed by 6.3%, increased URM interviews conducted by 8.5%, and resulted in an overall 19.2% increase in URM candidates matriculated [14]. These findings suggest that committing to increasing diversity by actively implementing a holistic review process when assessing applicants is an effective way of increasing the number of underrepresented minorities in the program.

Fellowship directors held fellowship programs most responsible for increasing diversity, followed by residency programs and medical schools. These responses suggest that directors of cardiology fellowship programs believe that increasing representation in cardiology requires the cooperation of administrators in undergraduate medical education as well as post-graduate training. Crowley et al. takes this argument a step further and encourages program directors to diversify the field through the development of a “deep pipeline” recruitment model through a partnership with local universities, high schools, and even elementary schools to recruit URMs to cardiology through early exposure to the field as a potential future career option [11]. Furthermore, the role of department chairs in promoting a culture of inclusion and representation amongst fellows and faculty and the responsibility to hire a diverse group of faculty is seen as important to achieving these goals by fellowship directors. Fellowship directors allocated considerably less responsibility to external organizations such as the ACGME, minority organizations (e.g., National Hispanic Medical Association), and physician professional organizations (e.g., American College of Cardiology), and the federal government. It may be that fellowship directors are not in favor of, are not aware of, or do not perceive additional involvement by the ACGME, government, or other regulatory agencies as effective in achieving meaningful growth in URM representation. In addition, attending physicians and applicants themselves were not held particularly responsible for increasing diversity, perhaps due to their limited role in affecting the composition of fellowship training programs.

This study adds to the literature in two ways: (1) It informs cardiology fellowship program directors of what many of their colleagues believe increases diversity in their programs as well as which strategies they implemented. These strategies may offer options for program directors seeking to increase diverse applicants. (2) These results may inform future research to identify which strategies statistically significantly increase the number of applicants and residents that are underrepresented minorities. In particular, prospective studies are needed with clearly defined metrics to track the success of initiatives to increase the number of URM applicants and eventual residents.

Limitations

Our study is not without limitations. Despite not using a previously validated survey instrument, we employed a robust developmental process that included review by content experts, presentations of the survey tool at local conferences to invite methodological critiques, and appraisal by associate cardiology program directors. Although our response rate was 28.4%, we used several strategies to drive responses, such as sending reminder emails and offering an optional lottery incentive. Notably, although we received responses from programs from different community settings and geographic locations, we were unable to compensate for non-response bias in this study, in which participants who responded to the disseminated survey may have disproportionately been interested in achieving increased racial and ethnic diversity within their programs compared to non-respondents. Also, this study was not designed to investigate female representation in cardiology fellowships, although women remain underrepresented in cardiology sub-specialty training [15]. Therefore, identifying methods to increase the proportion of women in cardiology fellowship training programs constitutes one area of future research.

## Conclusions

This cross-sectional study of cardiology fellowship program directors found that program directors (1) overwhelmingly supported increasing diversity in cardiology fellowship programs, (2) endorsed multiple strategies to increase diversity within their programs, and (3) believed that fellowship programs were most responsible for increasing diversity in the subspecialty of cardiology. Additionally, this study was able to quantify the amount of URM representation in ACGME accredited cardiology programs and identified that a majority of cardiology programs have URM faculty in leadership roles, and nearly a quarter of the surveyed program directors were URMs themselves. Interestingly, program directors held fellowship training programs most responsible for increasing URM representation in the field of cardiology. It is our hope that the results of this study may inform current cardiology fellowship program directors of which interventions are being used in other programs, which strategies are most supported by their peers, and which initiatives may yet need to be implemented. These findings may also be of value to medical students and resident physicians interested in applying to cardiology fellowships. Future research is needed to determine which strategies are most effective to increase URMs in cardiology fellowship programs in the United States.

## Appendices

Exhibit 1. Questionnaire for Cardiology Fellowship Program Directors

*URM = Under-Represented Minority: Any individual whose racial or ethnic identity is: Hispanic, Black, American Indian, Native Alaskan, Native Hawaiian, or Native Pacific Islander

1. What is the region of your residency program?

West

Southwest

Midwest

Southeast

Northeast

2. What category best describes your fellowship program?

Community

University

Hybrid

3. What is the location of your fellowship program?

Rural

Suburban

Urban

4. What is the length of your fellowship program in years?

5. How many total positions are available in your entire fellowship program?

6. How many total URM fellows are in your program across all positions?

7. How many URM fellows have you matriculated this year?

8. How many total applicants are interviewed for a fellowship position on average?

10-20

20-30

30-40

40-50

>50

9. How many URM applicants are interviewed for a fellowship position on average each year?

0-1

2-3

4-5

>6

10. How many URM faculty members are present in your program?

0-1

2-3

4-5

>6

11. Do any URM faculty hold leadership positions in your program?

Yes

No

Unsure

12. Are you an URM?

Yes

No

13. To what degree do you agree or disagree with the following statement: diversity is important to my fellowship program.

Strongly disagree

Somewhat disagree

Neither agree nor disagree

Somewhat agree

Strongly agree

14. Which of the following methods do you believe increase diversity in cardiology programs? Please select all that apply.

A). Outreach to URM Residents

                                               i.     Directly recruit URM to apply to your fellowship program

                                             ii.     Allow URM applicants the opportunity to connect with current URM fellows

                                            iii.     Directly contact medical schools that graduate large numbers or percentages of URMs for recruitment purposes

                                            iv.     Involve current program fellows in informal recruitment of URMs

                                             v.     Offer stipends and/or travel awards for URM residents interested in applying to your program

B). Altering fellowship application review of URM applicants

                                               i.     Deemphasize USMLE scores when reviewing applicants

                                             ii.     Conduct holistic review of applicants

                                            iii.     Remove applicant photos and ethnicities from applications

                                            iv.     Require acceptance of a minimum number of URM applicants

                                             v.     Expand the fellowship selection committee to include URM reviewers

                                            vi.     Consider acceptance of more international medical graduates

C). Discussion of diversity-related programming within your program or institution during interview day activities

                                               i.     Allocation of additional institutional funding and resources to diversity initiatives

                                             ii.     Attempt to increase the number of faculty members that are URM

                                            iii.     Create a small group session for URM and all other interested fellows to discuss workplace issues

                                            iv.     Formalize methods to surveil and address instances of micro-aggressions, discriminatory behavior, and abuse        against minorities

15. Which of the following methods do you currently implement in your cardiology program in order to promote diversity? Please select all that apply.

A). Outreach to URM Residents

                                               i.     Directly recruit URM to apply to your fellowship program

                                             ii.     Allow URM applicants the opportunity to connect with current URM fellows

                                            iii.     Directly contact medical schools that graduate large numbers or percentages of URMs for recruitment purposes

                                            iv.     Involve current program fellows in informal recruitment of URMs

                                             v.     Offer stipends and/or travel awards for URM residents interested in applying to your program

B). Altering fellowship application review of URM applicants

                                               i.     Deemphasize USMLE scores when reviewing applicants

                                             ii.     Conduct holistic review of applicants

                                            iii.     Remove applicant photos and ethnicities from applications

                                            iv.     Require acceptance of a minimum number of URM applicants

                                             v.     Expand the fellowship selection committee to include URM reviewers

                                            vi.     Consider acceptance of more international medical graduates

C). Discussion of diversity-related programming within your program or institution during interview day activities

                                               i.     Allocation of additional institutional funding and resources to diversity initiatives

                                             ii.     Attempt to increase the number of faculty members that are URM

                                            iii.     Create a small group session for URM and all other interested fellows to discuss workplace issues

                                            iv.     Formalize methods to surveil and address instances of micro-aggressions, discriminatory behavior, and abuse        against minorities

16. Who has responsibility to increase diversity in cardiology programs? [No responsibility / Some responsibility / Major responsibility]

ACGME (Associate Council for Graduate Medical Education)

Residency programs

Fellowship programs

Departmental chairs

Departmental physicians

Applicants

Medical schools

Physician professional organizations

Minority physician interest organizations

Federal government

17. Do you have any comments about diversity in fellowship programs or this study? (Optional)
